# Effect of Cryogenic Treatment on Low-Density Magnesium Multicomponent Alloys with Exceptional Ductility

**DOI:** 10.3390/ma19010100

**Published:** 2025-12-27

**Authors:** Yu Fang, Michael Johanes, Manoj Gupta

**Affiliations:** Department of Mechanical Engineering, National University of Singapore, 9 Engineering Drive 1, Singapore 117575, Singapore

**Keywords:** magnesium, lightweight materials, multicomponent alloys, cryogenic treatment, mechanical properties, disintegrated melt deposition

## Abstract

There is growing emphasis on lightweight and energy-efficient metallic materials, with multicomponent alloying (MCA) being one strategy to achieve this. This was combined with the inherently lightweight magnesium (Mg) as the base metal. Two Mg-based MCAs, namely Mg-71MCA and Mg-80MCA (Mg-10Li-9Al-6Zn-4Si and Mg-10Li-6Al-2Zn-2Si, respectively, wt.%), with density in the range of 1.55–1.632 g/cc akin to plastics were synthesized via the Disintegrated Melt Deposition method in this work. The effects of cryogenic treatment (CT) at –20 °C, 80 °C, and –196 °C (LN) on the physical, microstructural, thermal, and mechanical properties were systematically evaluated. CT resulted in densification, significant grain refinement (up to a 27.9% reduction in grain diameter after LN treatment), alterations in crystallographic texture, and notable changes to secondary phases—namely, an increased precipitate area fraction. These led to enhanced mechanical performance such as damping capacity, microhardness, and compressive response (most apparent for Mg-71MCA with 12.1%, 6.7%, and 1.6% increase in yield strength, ultimate compressive strength, and energy absorbed, respectively, after RF20 treatment), coupled with exceptional ductility (>80% strain without fracture), which is superior to pure Mg and commercial Mg alloys. Overall, this work showcases the potential of MCAs compared to existing conventional lightweight materials, as well as the property-enhancing/tailoring effects brought upon by different CT temperatures. This highlights the multi-faceted nature of material designs where compositional control and judicious processing parameter selection need to be both leveraged to optimize final properties, and serves as a baseline for further lightweight MCA development to meet future needs.

## 1. Introduction

Growing demand for lightweight and energy-efficient materials has stimulated intensive research into alternatives to conventional structural metals such as steel, aluminum, and titanium-based alloys [1]. Although these conventional metals exhibit excellent strength and durability, their high density and energy-intensive manufacturing processes pose high energy expenditures throughout their life cycles. Magnesium (Mg)-based alloys, with a low density (arising from that of its base metal at 1.74 g/cm^3^), high specific strength, and exceptional damping capacity, have emerged as promising candidates in transportation, consumer electronics, biomedical, and energy sectors [2]. Compared to steel (density ~7.86 g/cm^3^) and aluminum alloys (density ~2.7 g/cm^3^), Mg alloys can achieve component-level mass reductions of up to 75% and 33% when compared with equivalent steel and aluminum, respectively, which translates into an overall vehicle weight reduction of approximately 36% and fuel economy improvements of over 20%, to name automotives as just one example [3,4]. Mg alloys are also more sustainable as they exhibit high recyclability, with end-of-life recovery rates exceeding 90% under industrial conditions, and recycling consumes only ~5% of the energy required for primary production [5].

However, pure Mg and its binary alloys are not without compromises; they exhibit limited thermal stability and low ductility which restricts their use in demanding service environments. These inherent shortcomings must be addressed to enable broader structural applications [6]. Mg-based multicomponent alloys (MCAs) have the potential to improve strength through synergistic alloying but frequently contain brittle intermetallic compounds that degrade ductility. Therefore, achieving a balance between high strength and ductility remains a significant challenge [7].

Since Yeh et al. explored the multi-principal-element alloy design strategy in 2004 [8], these alloys have demonstrated hardness, strength, and wear resistance far exceeding those of traditional alloys [9,10]. Their exceptional performance is attributed to the high mixing entropy effect that stabilizes single-phase solid solutions, severe lattice distortion that hinders dislocation motion, and sluggish atomic diffusion that promotes nano-structured or even amorphous phases arising from multiple principal elements [11]. More recently, this approach has been applied to low-density systems by incorporating Mg as one of the principal elements, yielding lightweight Mg alloys which exhibit mechanical and thermal characteristics of their constituent elements [12,13]. Therefore, Mg-based MCAs represent a compelling pathway to next-generation structural materials combining light weight with superior mechanical and thermal properties [12].

The MCA strategy enables the tailoring of microstructures and phase distributions to achieve synergistic improvements in strength and toughness. Specifically, the incorporation of lithium (Li) significantly reduces alloy density and, at approximately 10 wt.%, triggers a phase transformation from hexagonal close-packed (HCP, normally found in Mg) to body-centered cubic (BCC), which activates additional slip systems to enhance ductility. Furthermore, the formation of metastable phases such as MgLi_2_ contributes to refined precipitate control, promoting an optimal balance between strength and ductility. However, excessive Li can lead to the formation of deleterious phases, reduced thermal stability, and diminished ignition resistance [14,15]. Other alloying elements include aluminum (Al) and zinc (Zn), which strengthen the matrix via solid solution strengthening and the precipitation of Mg_17_Al_12_ and MgZn phases, enhancing mechanical properties and high-temperature performance [6]. Trace additions of silicon (Si) promote grain refinement and improve wear resistance through the formation of fine Mg_2_Si precipitates [16].

Based on the potential MCAs have to offer, two lightweight Mg-based compositions—Mg–10Li–9Al–6Zn–4Si and Mg–10Li–6Al–2Zn–2Si (wt.%)—were studied in this work to explore the possibility of enhancing overall mechanical response while retaining the high ductility of Mg-Li systems.

To further exploit the advantages of MCAs beyond compositional control, this work also explores cryogenic treatment (CT) at three different sub-zero temperatures: −20 °C, −80 °C, and −196 °C (liquid nitrogen immersion). This exploration was prompted by observations that differing sub-zero temperatures resulted in varying effects on material properties in past works found in the literature. For instance, exposure to −20 °C relieves residual stresses and slightly refines grains [17]. At −196 °C, dislocation density rises sharply and additional phase transformations can occur, leading to further gains in mechanical properties [18,19,20].

This work systematically examines how compositional control and CT influence microstructure, thermal, and mechanical response, providing insights for designing next-generation high-performance lightweight Mg-based materials.

## 2. Materials and Methods

### 2.1. Synthesis

Two Mg-based MCAs are synthesized in this work, outlined in Table 1.

Mg turnings with a purity > 99.9% (Acros Organics, Morris Plains, NJ, USA) were used as the base material for these alloys. Li granules with a purity of 99.999% (Alfa Aesar Haverhill, MA, USA), Al lumps with a purity > 99.9% (B & S Aircraft Alloys Inc., Syosset, NY, USA), Zn shots with 99.99% purity (Alfa Aesar, Haverhill, MA, USA), and Si granules with a purity of 99.999% (Alfa Aesar, Haverhill, MA, USA) were added as alloying elements.

The materials were synthesized using the Disintegrated Melt Deposition (DMD) method at a target superheat temperature of 700 °C. The Li granules were pre-wrapped in Al foil to minimize Li loss by delaying their direct exposure to higher temperatures, while an argon (Ar) atmosphere was maintained during the process as a protective measure against oxidation [21]. Once the target temperature was reached, the melt was stirred with an impeller at 450 rpm for five minutes before being deposited into a mold to form ingots of 40 mm diameter.

The ingots were machined on a conventional lathe to billets approximately 35.5 mm in diameter and 45 mm in length. The billets were then coated with colloidal graphite to protect against oxidation and placed in a 10 mm diameter extrusion die (previously coated with the same colloidal graphite for lubrication) heated to 350 °C for 5 min before being hot-extruded. Samples were then cut from the resulting extruded rod and studied in the as-extruded (AE) form (without further processing, as well as after being subjected to CT at −20 °C, −80 °C, and −196 °C, immersed in liquid nitrogen, LN) for 24 h as outlined in Table 2. AE materials were used as reference for characterization results of materials which underwent CT. Where applicable, comparisons before and after treatment were conducted.

### 2.2. Physical Characterization

#### Density and Porosity

The theoretical density of the materials was calculated using the rule of mixtures. To calculate the experimental density, the Archimedes method was employed using AD-1653 density determination kit and a GH-252 electronic scale. Density measurements were conducted on five representative samples for each material, both before and after CT.

Each material was also subjected to elemental analysis. For Mg-71MCA, the sample was digested in a microwave at 240 °C for 15 min using a mixture of HNO_3_/HCl/HF in a 1:3:1 ratio. After cooling, 2 mL of 5% H_3_BO_3_ was added, and the sample was further digested in the microwave at 240 °C for an additional 15 min. For Mg-80MCA, the sample was digested on a hotplate at 240 °C for 15 min using aqua regia (HNO_3_/HCl in a 1:3 ratio), and the solution was then diluted to 15 mL with deionized water. The resulting liquids were characterized using a Perkin Elmer Avio 500 Inductively Coupled Plasma-Optical Emission Spectrometer (ICP-OES).

### 2.3. Microstructure

#### 2.3.1. General Microstructure

For microstructural analysis, samples from each material were first ground flat and then polished using a 0.05 µm alumina suspension. Microstructure images were subsequently obtained with an FESEM (Field Emission Scanning Electron Microscope, Hitachi S-4300, Hitachi, Ltd., Tokyo, Japan) equipped with EDS (Energy Dispersive Spectroscopy).

Polished sample surfaces were etched using a 2% Nital (2% HNO_3_ and 98% ethanol) for 2–3 s to reveal grains for analysis. Grain images were taken, and morphologies were analyzed using MATLAB (version R2013b, The MathWorks, Inc., Natick, MA, USA) in accordance with ASTM E112-13 (2021) [22].

#### 2.3.2. Secondary Phase Analysis

The secondary phases were quantified by applying a color-threshold segmentation to the SEM micrographs using ImageJ (version 1.54m, National Institutes of Health, Bethesda, MD, USA), and subsequent area fraction measurements of the predominant Mg_2_Si phase were conducted in MATLAB (version R2013b).

#### 2.3.3. X-Ray Diffraction

The longitudinal surfaces of the materials (perpendicular to the extrusion direction) were analyzed using X-Ray Diffraction (XRD) with a Shimadzu XRD-6000 automatic spectrometer (Shimadzu Corporation, Kyoto, Japan), utilizing Cu K(α) X-rays. The scan range (2θ, where θ is the Bragg angle) was set from 10° to 80° with a scanning rate of 2° per minute.

### 2.4. Thermal Characterization

Ignition response of the materials investigated was determined using thermogravimetric analysis (TGA). Samples of approximately 15 mg were placed in a crucible and analyzed using a Shimadzu DTG-60H thermogravimetric analyzer. The samples were heated from 30 °C to 1400 °C at a constant rate of 10 °C per minute in purified air, with a flowrate maintained at 50 mL/min.

To characterize the thermal response, a Shimadzu DSC-60 calorimeter was used on samples similar to those used for ignition response. The temperature range was 30 °C to 600 °C at a rate of 5 °C per minute in argon gas (25 mL/min flowrate).

Samples approximately 5–6 mm in length were ground flat and tested using the automated TMA PT1000 thermo-mechanical analyzer. The samples were heated from 50 °C to 400 °C at a rate of 5 °C per minute in argon gas at a flow rate of 0.1 L/min to measure the coefficient of thermal expansion (CTE).

### 2.5. Mechanical Characterization

#### 2.5.1. Damping Response

**Figure 1:**
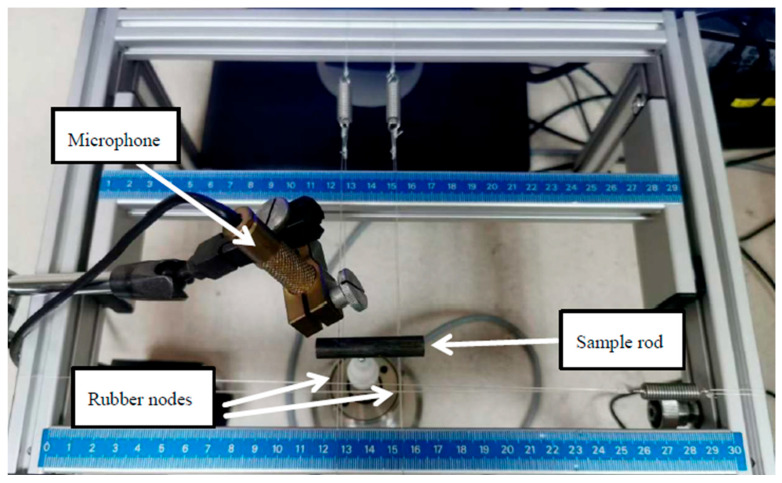
Damping characterization setup.

To determine the damping properties of the materials, samples of approximately 50 mm in length were subjected to impulse excitation. The resulting vibration signals were analyzed using Resonance Frequency Damping Analyzer (RFDA) software, version 8.1.2. Figure 1 provides an image of the experimental setup.

#### 2.5.2. Hardness

Polished sample surfaces were characterized using a Shimadzu HMV-2 automatic hardness tester. This was conducted in accordance with ASTM standard E384-16 [23], using a load of 980.7 mN with a dwell time of 15 s. A minimum of 15 readings was taken for each material.

#### 2.5.3. Compression

Flat and parallel samples from each material with an aspect ratio (L/D) of 1 underwent quasi-static compression testing in accordance with ASTM E9-09 [24]. The tests were performed using an automatic servo-hydraulic testing system (MTS 810). The test speed was set at 0.5% of the original sample length per minute (0.0083% s^−1^), with each sample compressed to 20% of its initial length or until failure, whichever was earlier. A minimum of 3 samples was characterized to obtain representative results.

## 3. Results and Discussion

### 3.1. Density and Porosity

The measured elemental compositions of the synthesized Mg-MCAs are outlined in Table 3, showing minimized Li loss.

Table 4 outlines the theoretical and experimental densities of Mg-71MCA and Mg-80MCA under different CTs. The theoretical density was obtained by applying the rule of mixtures (which assumes no ordered phases) to the measured elemental composition obtained previously. The experimental density was found to be higher than the theoretical density due to the formation of (ordered) secondary phases in the alloy [25]. Furthermore, it is worth noting that the density range exhibited by these MCAs (1.550–1.632 g/cc) is much closer to those of plastics (e.g., ET, PVC, and PBT, which are near 1.500 g/cc) [26], significantly less than most Mg- and Al-based alloys.

A slight increase in density was observed after CT. This suggests that CT helps reduce processing-induced microvoids and improves the alloy’s densification [1].

### 3.2. Microstructure

**Figure 2:**
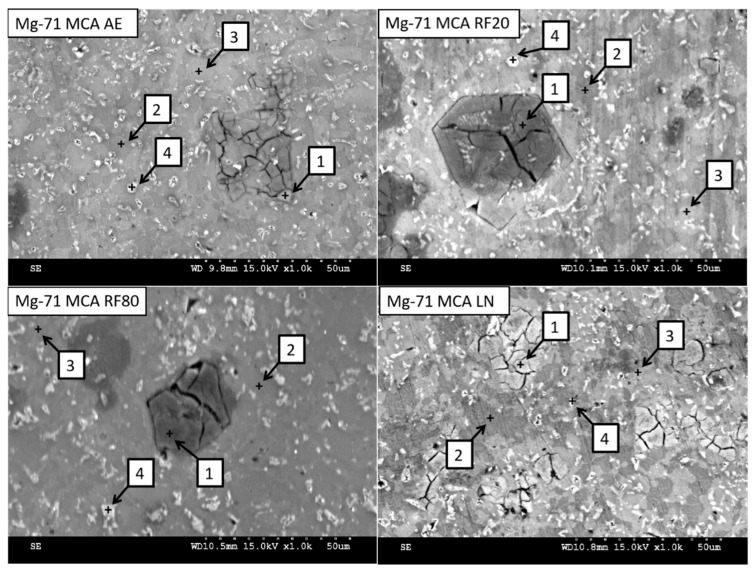
SEM micrographs of Mg-71MCA materials, with selected spectra locations.

**Figure 3:**
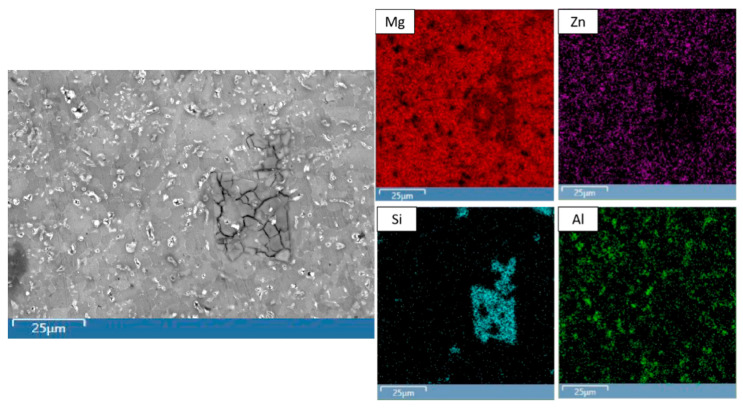
Representative mapping results of Mg-71MCA.

Li was not detected since EDS is unable to detect elements with low atomic radii (atomic number < 4). Figure 2 and Table 5 show the microstructure and EDS results of selected areas within the Mg-71MCA microstructure, and Figure 3 shows the results of Mg-71MCA mapping. Most of the microstructure consists of a Mg matrix, but the dark regions (spectrum 2) and bright regions (spectrum 3) are evident in SEM micrographs. Mapping and EDS results show that bright regions contain more Al, the region where the distinct crack network is clearly seen (spectrum 1). Mapping and EDS show this region is predominantly composed of Mg and Si, which likely contains the Mg_2_Si intermetallic phase corresponding to the weight fraction of Si in this MCA [27]. In addition, there are a small number of bright particles containing elevated levels of Zn and Al (spectrum 4).

**Figure 4:**
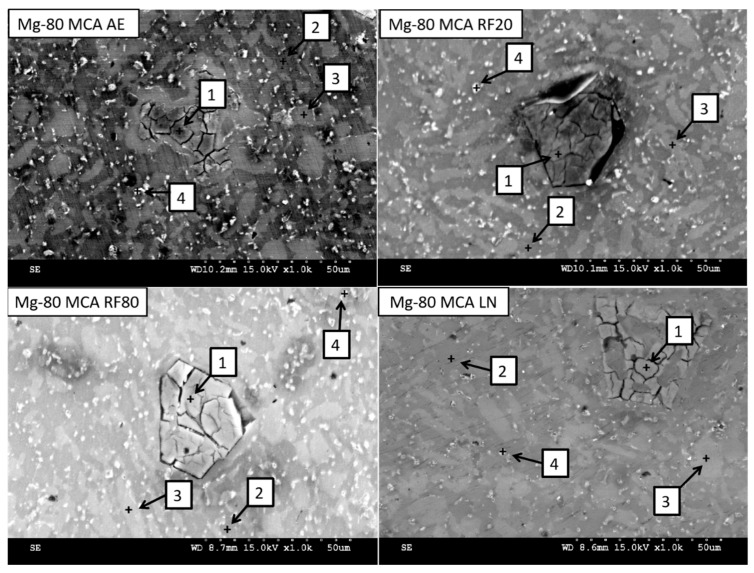
SEM micrographs of Mg-80MCA materials, with selected spectra locations.

**Figure 5:**
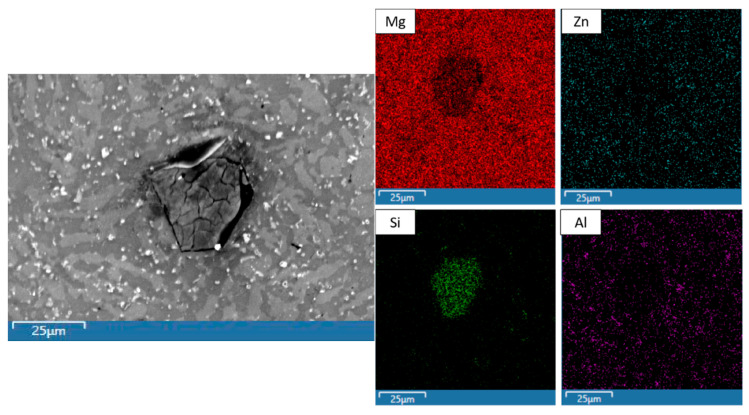
Representative mapping results of Mg-80MCA.

Figure 4 and Table 6 show the microstructure and EDS results of selected areas within the Mg-80MCA microstructure, and Figure 5 shows the results of Mg-80MCA mapping. Much like the Mg-71MCA characterized previously, most of the microstructure consists of a Mg matrix, which shows clearly dark regions (spectrum 2) and bright regions (spectrum 3) in SEM micrographs. However, there are not significant differences in the elemental compositions between the dark and bright regions in this case. Spectrum 1 (where the distinct crack network is seen) most likely contains the Mg_2_Si intermetallic phase due to the levels of Mg and Si detected, which corresponds to the weight fraction of Si in this MCA [27]. Mg-80MCA has a small number of bright particles (spectrum 4) with Al and Zn content, as was previously seen in Mg-71MCA.

#### 3.2.1. Secondary Phase Characterization

Secondary phase data are outlined in Table 7. In Mg-71MCA, RF20 and LN treatments increased the area fraction by 47.2% and 64.6%, respectively, while RF80 induced a 26.8% coarsening in the average precipitate diameter, indicating that sub-zero exposures significantly promote intermetallic precipitation and growth. This increase in precipitate area fraction can be attributed to the formation of additional nucleation sites resulting from volumetric contraction and the associated rise in compressive stresses and stored deformation energy following CT [28]. Secondary phase area fraction was instead reduced for Mg-80MCA after RF20 and RF80 treatment (by 10.5% and 14.4%, respectively), with only LN treatment increasing precipitation. This highlights the importance of process parameter selection in the resulting microstructure. Further work is continuing in this area.

#### 3.2.2. X-Ray Diffraction

JCPDS card numbers from the Powder Diffraction File (PDF-5+, 2024) [29] were checked against the observed XRD peaks: 00-004-0770 for Mg, 00-004-0829 for MgO, 00-035-0773 for Mg_2_Si, 00-015-0228 for Mg_2_AlZn, 00-040-1334 for MgZn, 00-072-0536 for MgZn_2_, 04-019-0641 for MgLiZn, and 04-014-7592 for Mg_17_Al_12_.

**Figure 6:**
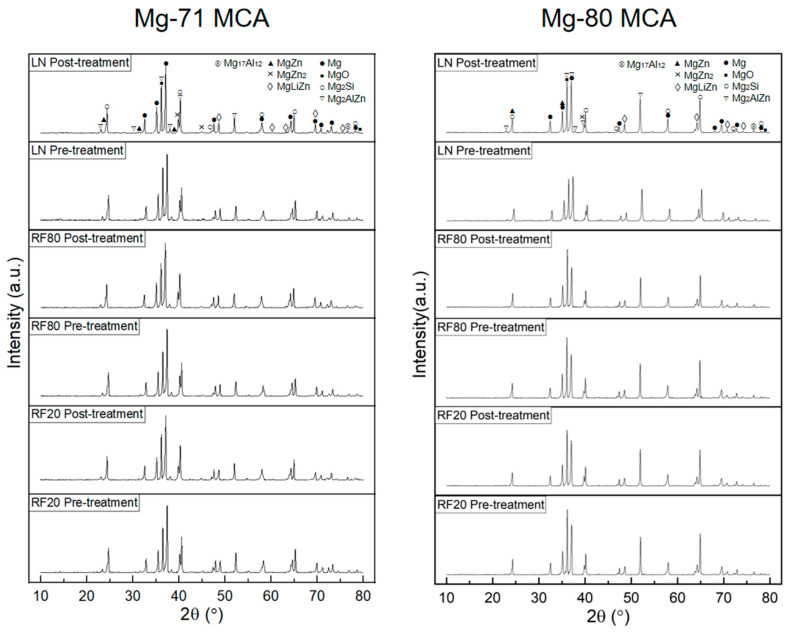
XRD diffractograms of Mg-MCAs in this work.

Figure 6 displays the XRD plots for Mg-MCAs in this work. The dominant diffraction peaks correspond to the α-Mg phase, confirming that α-Mg is the primary matrix. In addition, other peaks corresponding to secondary phases such as Mg_2_Si, Mg_2_AlZn, MgZn, MgZn_2_, MgLiZn, and Mg_17_Al_12_ are detected. Based on these findings, XRD further supports the findings observed within the microstructure (presence of Mg_2_Si and Mg_2_AlZn intermetallic phases, consistent with the alloying elements added [30]).

From Table 8, changes in the texture of Mg crystallographic planes were observed after CT; though the pyramidal texture remained dominant across all materials before and after CT, RF20-treated materials exhibited a slight increase in relative intensity for the basal texture. In contrast, exposure to LN reduced said relative intensity.

With regards to absolute intensity, CT temperature also had differing effects, with RF20 reducing the absolute intensity of the materials after treatment, and LN treatment imparting the opposite effect.

Both of these observations suggest that CT results in exposure temperature-dependent effects on the texture of the materials [31].

#### 3.2.3. Grain Characterization

Table 9 summarizes grain morphology information. CT resulted in noticeable grain refinement (in excess of 25% with LN exposure), consistent with previous studies on the effects of such treatment on Mg-based alloys [32,33]. This may be attributed to differential thermal contraction between different phases and grains of various orientations; under deep CT, both the matrix and the precipitate phases are predicted to undergo severe plastic deformation. This deformation is expected to lead to low-temperature dynamic recrystallization of the matrix as well as refinement of the precipitate hardening dispersion [34]. No significant change was observed in aspect ratio, which was observed with cryogenic-treated Mg/2 wt.%CeO_2_ [35].

### 3.3. Thermal Response

**Figure 7:**
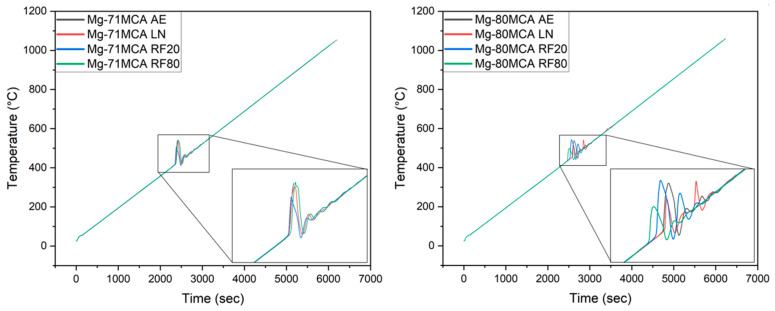
Ignition response of Mg-MCAs in this work.

**Figure 8:**
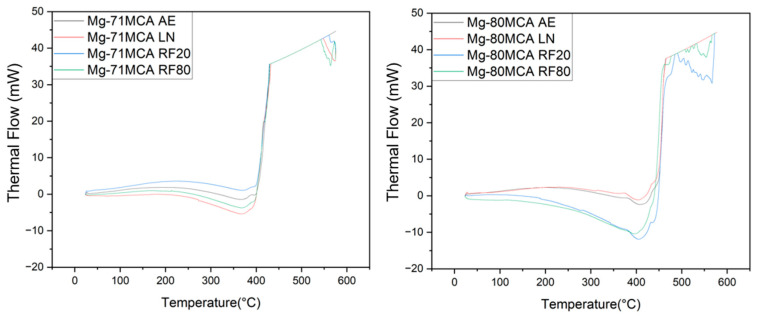
Thermal response of Mg-MCAs in this work.

Figure 7 and Figure 8, as well as Table 10, outline the thermal response of Mg-MCAs. The ignition temperature of both materials ranges from 417 °C to 459 °C, significantly lower compared to approximately 623 °C for pure Mg [36] and about 580–590 °C for other Mg alloys such as AZ91 [37]. The relatively low ignition temperatures observed in these materials is attributed to the high Li content in the Mg-MCAs; premature oxidation of Li relative to Mg initiates the reaction of Mg-Li phases with oxygen [38].

No significant change in ignition response with CT was observed for Mg-71MCA, consistent with previous studies on the effects of low-temperature treatment on Mg–2Zn–1Ca–0.3Mn alloy [39].

In contrast, for Mg-80MCA, LN treatment results in a noticeable reduction in ignition temperature (by 4.4%). This is attributed to an increased secondary phase area fraction which results in a more oxidation-prone surface owing to the presence of Li in some of the phases.

For Mg-71MCA, a distinct exothermic peak is observed around 420 °C, whereas for Mg-80MCA, a clear exothermic peak appears around 450 °C. This demonstrates a good consistency between the thermal responses observed by TGA and DSC, confirming that the pronounced exothermic peaks correspond to oxidation/ignition, while the endothermic trend above 500 °C indicates the partial melting/softening of alloy constituents well below Mg’s melting point of 660 °C.

While there were subtle microstructural changes, such as reduced porosity or minor defect rearrangements that slightly diminish lattice expansion, no significant changes in CTE were noted for Mg-71MCA except for RF80 exposure when CTE reduced by 8.7% [40].

Moderate CT (RF80) resulted in the most significant change in CTE for Mg-80MCA due to the introduction of additional lattice strain and defects, while deep CT (LN) lowered CTE by reducing lattice expansion (strengthening the matrix against the propagation of existing defects and lowering porosity) [41].

### 3.4. Mechanical Characterization

#### 3.4.1. Damping Response

The damping properties of Mg-MCAs under different CTs in this work are summarized in Table 11. The frequency response curves can be plotted by equations in the form y = Ae^−kx^, where k is the attenuation coefficient.

Compared to pure Mg which has an attenuation coefficient of 5.7 [42], the Mg-MCAs in this work exhibit attenuation coefficients ranging from 27.5 to 38, indicating significantly enhanced damping performance. Additionally, the Mg-MCAs explored in this study demonstrated improved stiffness over that of pure Mg (E = 44 GPa).

Across both Mg-MCAs, RF20 treatment improves both the attenuation coefficient and damping capacity (i.e., 16.6% and 7.6%, respectively, for Mg-71MCA; 27.7% and 20.3%, respectively, for Mg-80MCA). This improvement may be attributed to microstructural refinement and reduced residual stress induced by CT [40]. However, lower CT temperatures (RF80 and LN) compromised damping performance, attributed to decreased porosity and reduction in voids which would otherwise act as air pockets and concentrations of local stress as well as plastic deformation [43,44,45,46], and effectively dissipate energy in addition to dislocation-related effects normally present in dense metallic materials [47,48]. This highlights a potential compromise in performance with differing CT temperatures [49].

#### 3.4.2. Hardness

The Mg-MCAs explored in this study exhibited high hardness (Table 12), significantly higher than that of pure Mg (66 H_V_) [42]. This increase in hardness is mainly attributed to the strengthening effects of the alloying elements; the addition of Li, Al, Zn, and Si not only promotes the formation of secondary intermetallic phases but also contributes to solid-solution strengthening [50]. LN treatment was most impactful, with 113 Hv and 93 Hv (8.7% and 9.4% increases) exhibited by Mg-71MCA and Mg-80MCA, respectively, which are superior to conventional/commercial Mg alloys such as AZ31 (60–85 H_V_) [51] and AZ91 (80–105 H_V_) [20].

Several interrelated factors induced by sub-zero temperature exposure can account for this. First, the increase in dislocation density provided additional obstacles to dislocation motion [31,52]. Second, an increased intensity of basal planes at the surface was observed, which contributed to the enhanced microhardness [19]. Induced compressive stresses also strain the lattice and inhibit plastic deformation [17,35]. The Hall–Petch effect also comes into play due to grain refinement; the increase in grain boundary area further impedes dislocation movement [53]. Finally, the increased area fraction of uniformly distributed, fine secondary phases contributes directly to increased hardness [54].

#### 3.4.3. Compressive Response

**Figure 9:**
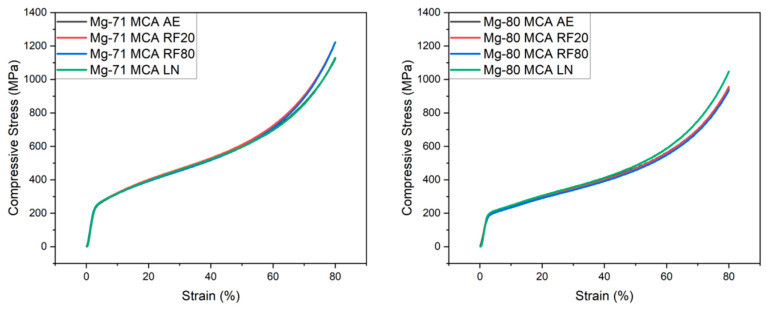
Representative compressive stress–strain curves of Mg-MCAs in this work.

Figure 9 and Table 13 outline compressive response. No materials underwent fracture at up to 80% strain. This exceptional ductility is primarily attributed to the addition of Li which activates non-basal slip systems [55]; it also promotes the formation of the β-Li phase which exhibits high deformability at room temperature owing to its BCC structure [56].

CT enhanced the yield strength by approximately 12% for Mg-71MCA, with additional improvements in ultimate compressive strength (UCS) as well (6.7% increase with RF20 treatment). This enhancement is largely attributed to the intrinsic microstructural factors that govern friction stress and dislocation motion, which in turn affect yield strength and evolve differently under various low-temperature exposures [35]. Moreover, the observed grain refinement contributes to the improved compressive response in accordance with the Hall–Petch relationship [53]. These improvements were similarly observed in studies on AZ91 Mg-based alloys [41]. A further contributing factor to mechanical enhancement is the decreased porosity brought upon by CT.

However, the same improvements were not realized in Mg-80MCA. Only LN exposure resulted in compressive response improvements, in line with literature results on LN exposure of Mg-materials [41]. Instead, minor property degradation was exhibited after moderate CT (RF20 and RF80). Slight weakening of the basal texture was observed from the XRD results (Table 8) in absolute terms, by 9.6% and 21.1% after RF20 and RF80, respectively. As the basal texture is responsible for higher strength, such texture changes led to modest decreases in yield strength, ultimate compressive strength, and energy absorption, reflected in Mg-80MCA RF80 experiencing greater compromise in compressive properties [51,57,58]. Overall, LN CT provides the best combined compressive properties.

## 4. Conclusions

Two Mg-based MCAs were successfully synthesized and subjected to CT at −20 °C, −80 °C, and −196 °C (LN exposure). The following key conclusions can be drawn up.

1. Mg-MCAs exhibit densification after CT, with maximum increases of 0.31% for Mg-71MCA RF80 and 0.19% for Mg-80MCA RF20).

2. CT refines the average grain size in Mg-MCAs, with maximum reductions of 26.6% for Mg-71MCA and 27.9% for Mg-80MCA as treatment temperature decreases, while the aspect ratio remains essentially unchanged.

3. Ignition resistance of these MCAs was compromised (auto-ignition temperatures of 460 °C and below), but were mostly unaffected by CT. In addition, for Mg-71MCA, CTE generally remains unchanged after CT. Mg-80MCA shows a slight increase in CTE under moderate treatment (up to 16.1% with RF80).

4. Moderate CT enhances damping capacity (by up to 16.6% for Mg-71MCA and 27.7% for Mg-80MCA), whereas ultra-low temperature treatment (LN) compromises it (by 18.8% and 9.8%, respectively). Young’s modulus is slightly enhanced by CT, with increases of 3.5% for Mg-71MCA (RF20) and 1.8% for Mg-80MCA (LN). CT affects microhardness positively, reaching 113 H_V_ for Mg-71MCA (an 8.7% improvement) and 93 H_V_ for Mg-80MCA (a 9.4% improvement) after LN treatment.

5. Mg-71MCA exhibited a 12.1%, 6.7%, and 1.6% increase in yield strength, UCS, and energy absorbed, respectively, after RF20 treatment. Mg-80MCA’s gains were most apparent after LN treatment (increases of 3.6%, 5.9%, and 5.1% for the same properties), with Mg-71MCA as the superior load-bearing material. Exceptional ductility was the key feature of the two MCAs, which remained unaffected by CT irrespective of changes in mechanical response.

6. Overall, for Mg-71MCA, mild cryogenic treatment (−20 °C) is recommended for optimal strength, hardness, and damping performance. For Mg-80MCA, ultra-low-temperature (LN) treatment is optimal, as it greatly improves mechanical strength and hardness with a slight, acceptable reduction in damping performance.

The findings highlight the potential for such lightweight alloys in structural use (combining low density (<1.632 g/cc), good strength, and exceptional ductility (>80%)), with Mg-71MCA positioned as the optimal composition due to low weight (as low as 1.550 g/cc) and enhanced mechanical properties (in particular, yield strength), making it most suitable for weight-sensitive applications without exposure to high temperatures (e.g., structural components in high-performance automotives).

At the same time, the effect of cryogenic temperature is highlighted with different impacts on different properties; the lowest temperature may not always be the optimal processing route.

## Figures and Tables

**Table 1:** Elemental compositions of Mg-MCAs explored in this work.

Material Designation	Composition (wt.%)	Elemental Composition (wt.%)					Total (%)
		Mg	Li	Al	Zn	Si	
Mg-71MCA	Mg-10Li-9Al-6Zn-4Si	71	10	9	6	4	100
Mg-80MCA	Mg-10Li-6Al-2Zn-2Si	80	10	6	2	2	100

**Table 2:** Summary of processing conditions and material designation suffix.

Condition	Material Designation Suffix
As-extruded, no further treatments	AE
Refrigerated at −20 °C for 24 h	RF20
Refrigerated at −80 °C for 24 h	RF80
Immersed in liquid nitrogen at −196 °C for 24 h	LN

**Table 3:** Measured material composition of Mg-MCAs in this work.

Material	Measured Elemental Composition (wt.%)				
	Mg	Li	Al	Zn	Si
Mg-71MCA	70.44	10.88	9.36	6.87	2.45
Mg-80MCA	80.29	10.18	6.36	1.85	1.32

**Table 4:** Density and porosity results of the Mg-based materials, before and after CT.

Material	Condition	Theoretical Density (g/cm^3^)	Experimental Density (g/cm^3^)
Mg-71MCA RF20	Pre-treatment	1.503	1.624 ± 0.001
	Post-treatment		1.627 ± 0.003 (↑ 0.18%)
Mg-71MCA RF80	Pre-treatment		1.627 ± 0.001
	Post-treatment		1.632 ± 0.004 (↑ 0.31%)
Mg-71MCA LN	Pre-treatment		1.626 ± 0.002
	Post-treatment		1.629 ± 0.010 (↑ 0.18%)
Mg-80MCA RF20	Pre-treatment	1.458	1.552 ± 0.004
	Post-treatment		1.555 ± 0.005 (↑ 0.19%)
Mg-80MCA RF80	Pre-treatment		1.551 ± 0.004
	Post-treatment		1.552 ± 0.003 (↑ 0.06%)
Mg-80MCA LN	Pre-treatment		1.554 ± 0.003
	Post-treatment		1.550 ± 0.003 (↓ 0.26%)

**Table 5:** EDS results for Mg-71MCA in this work.

Material	Spectrum	Detected Element (wt.%)			
		Mg	Al	Zn	Si
Mg-71MCA AE	1	48.59	1.49	1.38	48.54
	2	78.83	3.92	17.04	0.21
	3	77.67	8.16	13.85	0.32
	4	73.78	7.68	18.11	0.43
Mg-71MCA RF20	1	50.89	2.65	4.55	41.92
	2	80.03	5.15	13.97	0.86
	3	75.75	7.52	15.77	0.95
	4	45.30	21.77	32.27	0.66
Mg-71MCA RF80	1	51.91	1.93	2.47	43.69
	2	83.39	4.19	11.43	0.98
	3	64.02	12.02	23.45	0.51
	4	53.41	18.77	26.12	1.70
Mg-71MCA LN	1	53.06	0.52	1.25	45.18
	2	83.26	1.81	14.83	0.10
	3	85.71	4.60	8.61	1.09
	4	30.29	27.71	41.51	0.49

**Table 6:** EDS results for Mg-80MCA in this work.

Material	Spectrum	Detected Element (wt.%)			
		Mg	Al	Zn	Si
Mg-80MCA AE	1	53.09	0.83	0.50	45.58
	2	90.17	3.29	6.43	0.11
	3	86.88	7.10	5.91	0.11
	4	35.49	27.92	30.71	5.88
Mg-80MCA RF20	1	51.67	1.41	0.79	46.13
	2	76.57	9.28	7.49	6.66
	3	75.79	5.44	4.74	14.03
	4	89.48	4.69	5.03	0.80
Mg-80MCA RF80	1	52.79	1.63	0.79	44.79
	2	87.31	6.19	6.05	0.45
	3	89.04	4.52	5.99	0.45
	4	89.28	4.78	5.26	0.68
Mg-80MCA LN	1	46.83	0.87	0.00	52.30
	2	92.89	3.28	3.71	0.11
	3	85.43	8.54	5.69	0.33
	4	87.85	5.03	7.00	0.11

**Table 7:** Secondary phase data of Mg-MCAs in this work.

Material	Condition	Secondary Phase Area Fraction (%)	Average Secondary Phase Diameter (µm)	Aspect Ratio
Mg-71MCA	AE	15.3	2.64 ± 0.87	2.02 ± 0.83
	RF20	22.6 (↑ 47.2%)	2.86 ± 1.08 (↑ 8.3%)	2.01 ± 0.84
	RF80	16.4 (↑ 7.0%)	3.35 ± 1.11 (↑ 26.8%)	2.06 ± 0.85
	LN	25.2 (↑ 64.6%)	3.04 ± 1.14 (↑ 15.2%)	1.98 ± 0.75
Mg-80MCA	AE	12.3	1.67 ± 0.81	1.99 ± 0.88
	RF20	11.0 (↓ 10.5%)	1.82 ± 0.81 (↑ 8.6%)	1.75 ± 0.64
	RF80	10.5 (↓ 14.4%)	1.75 ± 0.72 (↑ 4.5%)	1.90 ± 0.77
	LN	14.0 (↑ 13.8%)	1.71 ± 0.78 (↑ 2.5%)	2.07 ± 1.07

**Table 8:** Relative and absolute intensities of Mg crystalline plane peaks of Mg-MCAs.

Material	Crystal Plane	Relative Intensity (I/I_max_)		Absolute Intensity	
		Pre-Treatment	Post Treatment	Pre-Treatment	Post Treatment
Mg-71MCA RF20	10-10 Prismatic	0.2075	0.2233	249	203
	0002 Basal	0.3358	0.3454	403	314
	10-11 Pyramidal	1	1	1200	909
Mg-71MCA RF80	10-10 Prismatic	0.2045	0.2029	247	255
	0002 Basal	0.3667	0.3811	443	479
	10-11 Pyramidal	1	1	1208	1257
Mg-71MCA LN	10-10 Prismatic	0.2147	0.2199	146	316
	0002 Basal	0.3971	0.389	270	559
	10-11 Pyramidal	1	1	680	1437
Mg-80MCA RF20	10-10 Prismatic	0.2382	0.2251	257	219
	0002 Basal	0.4717	0.4728	509	460
	10-11 Pyramidal	1	1	1079	973
Mg-80MCA RF80	10-10 Prismatic	0.2341	0.2378	257	210
	0002 Basal	0.5537	0.5436	608	480
	10-11 Pyramidal	1	1	1098	883
Mg-80MCA LN	10-10 Prismatic	0.2334	0.228	225	251
	0002 Basal	0.4616	0.4269	445	470
	10-11 Pyramidal	1	1	964	1101

**Table 9:** Grain data of Mg-MCAs in this work.

Material	Condition	Average Grain Diameter (µm)	Aspect Ratio
Mg-71MCA	AE	4.03 ± 1.17	1.52 ± 0.36
	RF20	3.46 ± 1.05 (↓ 14.1%)	1.50 ± 0.33
	RF80	3.64 ± 1.23 (↓ 9.7%)	1.46 ± 0.43
	LN	2.96 ± 0.89 (↓ 26.6%)	1.45 ± 0.33
Mg-80MCA	AE	3.91 ± 1.11	1.40 ± 0.25
	RF20	3.54 ± 1.13 (↓ 9.5%)	1.36 ± 0.19
	RF80	3.65 ± 1.07 (↓ 6.7%)	1.50 ± 0.38
	LN	2.82 ± 1.03 (↓ 27.9%)	1.44 ± 0.25

**Table 10:** Auto-ignition temperatures and CTE of Mg-MCAs in this work.

Material	Condition	Ignition Temperature (°C)	Average CTE (10^−6^/K)
Mg-71MCA	AE	417	28.9 ± 3.2
	RF20	417	28.8 ± 3.1 (↓ 0.3%)
	RF80	416	26.4 ± 4.9 (↓ 8.7%)
	LN	420	28.9 ± 2.5 (↑ 0%)
Mg-80MCA	AE	456	26.6 ± 4.3
	RF20	459	28.4 ± 3.4 (↑ 6.6%)
	RF80	448	30.9 ± 2.4 (↑ 16.1%)
	LN	436	25.7 ± 5.6 (↓ 3.6%)

**Table 11:** Damping results of Mg-MCAs in this work.

Material	Condition	Attenuation Coefficient	Damping Capacity (×10^−4^)	E-Modulus (GPa)
Mg-71MCA RF20	Pre-treatment	27.50	4.84	53.09
	Post-treatment	32.08 (↑ 16.6%)	5.21 (↑ 7.6%)	54.97 (↑ 3.5%)
Mg-71MCA RF80	Pre-treatment	37.88	6.08	53.98
	Post-treatment	30.77 (↓ 18.8%)	4.86 (↓ 20.1%)	55.00 (↑ 1.9%)
Mg-71MCA LN	Pre-treatment	37.96	7.38	53.13
	Post-treatment	37.82 (↓ 0.4%)	6.90 (↓ 6.5%)	53.82 (↑ 1.3%)
Mg-80MCA RF20	Pre-treatment	26.58	4.92	52.08
	Post-treatment	33.93 (↑ 27.7%)	5.92 (↑ 20.3%)	51.85 (↓ 0.4%)
Mg-80MCA RF80	Pre-treatment	32.53	5.00	52.59
	Post-treatment	35.73 (↑ 9.8%)	5.87 (↑ 17.4%)	51.46 (↓ 2.1%)
Mg-80MCA LN	Pre-treatment	30.48	6.00	51.07
	Post-treatment	27.60 (↓ 9.4%)	5.18 (↓ 13.7%)	51.97 (↑ 1.8%)

**Table 12:** Microhardness and microhardness results of Mg-MCAs in this work.

Material	Condition	Average Microhardness (H_V_)
Mg-71MCA	AE	104 ± 4
	RF20	108 ± 5 (↑ 3.9%)
	RF80	112 ± 4 (↑ 7.7%)
	LN	113 ± 5 (↑ 8.7%)
Mg-80MCA	AE	85 ± 3
	RF20	90 ± 1 (↑ 5.9%)
	RF80	91 ± 3 (↑ 7.1%)
	LN	93 ± 2 (↑ 9.4%)

**Table 13:** Compressive response of Mg-MCAs in this work.

Material	Condition	Mean 0.2% Yield Strength (MPa)	Mean Ultimate Compressive Strength (MPa)	Mean Fracture Strain (%)	Mean Energy Absorbed (MJ/m^3^)
Mg-71MCA	AE	202 ± 6	1132 ± 30	>80	447 ± 4
	RF20	226 ± 13 (↑ 12.1%)	1208 ± 62 (↑ 6.7%)	>80	454 ± 7 (↑ 1.6%)
	RF80	226 ± 10 (↑ 11.9%)	1194 ± 66 (↑ 5.5%)	>80	455 ± 7 (↑ 1.9%)
	LN	227 ± 9 (↑ 12.7%)	1142 ± 20 (↑ 0.9%)	>80	445 ± 1 (↓ 0.4%)
Mg-80MCA	AE	176 ± 8	986 ±41	>80	354 ± 6
	RF20	165 ± 1 (↓ 6.5%)	967 ± 23 (↓ 2.0%)	>80	351 ± 2 (↓ 0.9%)
	RF80	164 ± 7 (↓ 7.0%)	944 ± 45 (↓ 4.3%)	>80	344 ± 6 (↓ 2.7%)
	LN	183 ± 2 (↑ 3.6%)	1044 ± 38 (↑ 5.9%)	>80	372 ± 5 (↑ 5.1%)

## Data Availability

The original contributions presented in this study are included in the article. Further inquiries can be directed to the corresponding author.

## References

[1] Gupta M., Wong W.L.E. (2015). Magnesium-based nanocomposites: Lightweight materials of the future. Mater. Charact..

[2] Song J., She J., Chen D., Pan F. (2020). Latest research advances on magnesium and magnesium alloys worldwide. J. Magnes. Alloys.

[3] Kulekci M.K. (2008). Magnesium and its alloys applications in automotive industry. Int. J. Adv. Manuf. Technol..

[4] Caceres C.H. (2007). Economical and Environmental Factors in Light Alloys Automotive Applications. Metall. Mater. Trans. A.

[5] Li R., Wang L., Yang B., Xu B., Liang D., Wang F., Tian Y. (2023). Magnesium Alloy Scrap Vacuum Gasification—Directional Condensation to Purify Magnesium. Metals.

[6] Yang Y., Xiong X., Chen J., Peng X., Chen D., Pan F. (2021). Research advances in magnesium and magnesium alloys worldwide in 2020. J. Magnes. Alloys.

[7] Yang Y., Xiong X., Chen J., Peng X., Chen D., Pan F. (2023). Research advances of magnesium and magnesium alloys worldwide in 2022. J. Magnes. Alloys.

[8] Yeh J.W., Chen S.K., Lin S.J., Gan J.Y., Chin T.S., Shun T.T., Tsau C.H., Chang S.Y. (2004). Nanostructured High-Entropy Alloys with Multiple Principal Elements: Novel Alloy Design Concepts and Outcomes. Adv. Eng. Mater..

[9] Zhang Y., Zhou Y.J., Lin J.P., Chen G.L., Liaw P.K. (2008). Solid-Solution Phase Formation Rules for Multi-component Alloys. Adv. Eng. Mater..

[10] Pickering E.J., Jones N.G. (2016). High-entropy alloys: A critical assessment of their founding principles and future prospects. Int. Mater. Rev..

[11] Yeh J.-W. (2006). Recent progress in high-entropy alloys. Eur. J. Control.

[12] Ma C., Hou C., Zhang X., Liu T., Zhou N., Zheng K. (2024). Study on the microstructure and mechanical properties of Mg–Al–Li–Zn–Ti multi-component alloy. J. Mater. Res. Technol..

[13] Johanes M., Bin Gombari A.A., Gupta M. (2023). Enhancing Multiple Properties of a Multicomponent Mg-Based Alloy Using a Sinterless Turning-Induced Deformation Technique. Technologies.

[14] Jin Z.-Z., Zha M., Wang S.-Q., Wang S.-C., Wang C., Jia H.-L., Wang H.-Y. (2022). Alloying design and microstructural control strategies towards developing Mg alloys with enhanced ductility. J. Magnes. Alloys.

[15] Li C., He Y., Huang H. (2021). Effect of lithium content on the mechanical and corrosion behaviors of HCP binary Mg–Li alloys. J. Magnes. Alloys.

[16] Liu M., Chen J., Lin Y., Xue Z., Roven H.J., Skaret P.C. (2020). Microstructure, mechanical properties and wear resistance of an Al–Mg–Si alloy produced by equal channel angular pressing. Prog. Nat. Sci. Mater. Int..

[17] Sonar T., Lomte S., Gogte C. (2018). Cryogenic Treatment of Metal—A Review. Mater. Today Proc..

[18] Singh Sidhu H., Singh B., Kumar P. (2021). Effect of cryogenic treatment on corrosion behavior of friction stir processed magnesium alloy AZ91. Mater. Today Proc..

[19] Dieringa H. (2017). Influence of Cryogenic Temperatures on the Microstructure and Mechanical Properties of Magnesium Alloys: A Review. Metals.

[20] Mónica P., Bravo P.M., Cárdenas D. (2017). Deep cryogenic treatment of HPDC AZ91 magnesium alloys prior to aging and its influence on alloy microstructure and mechanical properties. J. Mater. Process. Technol..

[21] XingHe T., Chee Keat How W., Chan Kwok Weng J., Kwok Wai Onn R., Gupta M. (2015). Development of high-performance quaternary LPSO Mg–Y–Zn–Al alloys by Disintegrated Melt Deposition technique. Mater. Des..

[22] (2021). Standard Test Methods for Determining Average Grain Size.

[23] (2017). Standard Test Method for Microindentation Hardness of Materials.

[24] (2018). Standard Test Methods of Compression Testing of Metallic Materials at Room Temperature.

[25] Song G., Atrens A. (2003). Understanding Magnesium Corrosion—A Framework for Improved Alloy Performance. Adv. Eng. Mater..

[26] Grigorescu R.M., Grigore M.E., Iancu L., Ghioca P., Ion R.-M. (2019). Waste Electrical and Electronic Equipment: A Review on the Identification Methods for Polymeric Materials. Recycling.

[27] Yan X.-Y., Chang Y.A., Zhang F. (2000). A thermodynamic analysis of the Mg-Si system. J. Phase Equilibria.

[28] Barylski A., Aniołek K., Dercz G., Kupka M., Kaptacz S. (2021). The effect of deep cryogenic treatment and precipitation hardening on the structure, micromechanical properties and wear of the Mg–Y-Nd-Zr alloy. Wear.

[29] Gates-Rector S., Blanton T. (2019). The Powder Diffraction File: A quality materials characterization database. Powder Diffr..

[30] Liang P., Tarfa T., Robinson J.A., Wagner S., Ochin P., Harmelin M.G., Seifert H.J., Lukas H.L., Aldinger F. (1998). Experimental investigation and thermodynamic calculation of the Al–Mg–Zn system. Thermochim. Acta.

[31] Jiang Y., Chen D., Chen Z., Liu J. (2010). Effect of Cryogenic Treatment on the Microstructure and Mechanical Properties of AZ31 Magnesium Alloy. Mater. Manuf. Process..

[32] Dong N., Sun L., Ma H., Jin P. (2021). Effects of cryogenic treatment on microstructures and mechanical properties of Mg-2Nd-4Zn alloy. Mater. Lett..

[33] Kumar S.D., Kumar S.S. (2023). Effect of Heat Treatment Conditions and Cryogenic Treatment on Microhardness and Tensile Properties of AZ31B Alloy. J. Mater. Eng. Perform..

[34] Li G.-r., Wang H.-m., Cai Y., Zhao Y.-t., Wang J.-j., Gill S.P.A. (2013). Microstructure and mechanical properties of AZ91 magnesium alloy subject to deep cryogenic treatments. Int. J. Miner. Metall. Mater..

[35] Gupta S., Parande G., Tun K.S., Gupta M. (2023). Enhancing the Physical, Thermal, and Mechanical Responses of a Mg/2wt.%CeO_2_ Nanocomposite Using Deep Cryogenic Treatment. Metals.

[36] Fassell W.M., Gulbransen L.B., Lewis J.R., Hamilton J.H. (1951). Ignition Temperatures of Magnesium and Magnesium Alloys. JOM.

[37] Ravi Kumar N.V., Blandin J.J., Suéry M., Grosjean E. (2003). Effect of alloying elements on the ignition resistance of magnesium alloys. Scr. Mater..

[38] Zhao W., Tang X., Li J., Le W., Jiao Q., Liu D., Jia Y. (2024). Atomized Mg-Li spherical alloys: A new strategy for promoting reactivity of Mg. J. Alloys Compd..

[39] Sole K., Johanes M., Gupta M. (2024). Enhancing Microstructural, Thermal, Mechanical, and Corrosion Response of a Bio/Eco-Compatible Mg–2Zn–1Ca–0.3Mn Alloy Using Two Types of Cryogenic Treatments. Adv. Eng. Mater..

[40] Johanes M., Mehtabuddin S., Venkatarangan V., Gupta M. (2024). An Insight into the Varying Effects of Different Cryogenic Temperatures on the Microstructure and the Thermal and Compressive Response of a Mg/SiO_2_ Nanocomposite. Metals.

[41] Asl K.M., Tari A., Khomamizadeh F. (2009). Effect of deep cryogenic treatment on microstructure, creep and wear behaviors of AZ91 magnesium alloy. Mater. Sci. Eng. A.

[42] Tekumalla S., Yang C., Seetharaman S., Wong W.L.E., Goh C.S., Shabadi R., Gupta M. (2016). Enhancing overall static/dynamic/damping/ignition response of magnesium through the addition of lower amounts (<2%) of yttrium. J. Alloys Compd..

[43] Xie Z.-k., Tane M., Hyun S.-k., Okuda Y., Nakajima H. (2006). Vibration–damping capacity of lotus-type porous magnesium. Mater. Sci. Eng. A.

[44] Li Q., Jiang G., Dong J., Hou J., He G. (2016). Damping behavior and energy absorption capability of porous magnesium. J. Alloys Compd..

[45] Golovin I.S., Sinning H.R. (2004). Internal friction in metallic foams and some related cellular structures. Mater. Sci. Eng. A.

[46] Golovin I.S., Sinning H.R., Arhipov I.K., Golovin S.A., Bram M. (2004). Damping in some cellular metalic materials due to microplasticity. Mater. Sci. Eng. A.

[47] Granato A., Lücke K. (1956). Theory of Mechanical Damping Due to Dislocations. J. Appl. Phys..

[48] Granato A., Lücke K. (1956). Application of Dislocation Theory to Internal Friction Phenomena at High Frequencies. J. Appl. Phys..

[49] Wang J., Zou Y., Dang C., Wan Z., Wang J., Pan F. (2024). Research Progress and the Prospect of Damping Magnesium Alloys. Materials.

[50] Polmear I., StJohn D., Nie J.-F., Qian M., Polmear I., StJohn D., Nie J.-F., Qian M. (2017). 6—Magnesium Alloys. Light Alloys.

[51] Che B., Lu L., Zhang J., Zhang J., Ma M., Wang L., Qi F. (2022). Effects of cryogenic treatment on microstructure and mechanical properties of AZ31 magnesium alloy rolled at different paths. Mater. Sci. Eng. A.

[52] Huang H., Zhang J. (2016). Microstructure and mechanical properties of AZ31 magnesium alloy processed by multi-directional forging at different temperatures. Mater. Sci. Eng. A.

[53] Hall E.O. (1951). The Deformation and Ageing of Mild Steel: III Discussion of Results. Proc. Phys. Soc. Sect. B.

[54] Hu J., Yang G., Liu Z., Fu S., Gao H. (2023). Effects of deep cryogenic treatment on microstructure, mechanical, and corrosion of ZK60 Mg alloy. J. Mater. Res. Technol..

[55] Xu J., Guan B., Xin Y., Huang G., Wu P., Liu Q. (2024). Revealing the role of pyramidal <c+a> slip in the high ductility of Mg-Li alloy. J. Magnes. Alloys.

[56] Shin I., Carter E.A. (2014). First-principles simulations of plasticity in body-centered-cubic magnesium–lithium alloys. Acta Mater..

[57] Hamad K. (2019). Highly-Ductile Magnesium Alloys: Atomistic-Flow Mechanisms and Alloy Designing. Materials.

[58] Suh B.-C., Shim M.-S., Shin K.S., Kim N.J. (2014). Current issues in magnesium sheet alloys: Where do we go from here?. Scr. Mater..

